# Research hotspots and trends of bone defects based on Web of Science: a bibliometric analysis

**DOI:** 10.1186/s13018-020-01973-3

**Published:** 2020-10-08

**Authors:** Haixiong Lin, Xiaotong Wang, Minling Huang, Zige Li, Zhen Shen, Junjie Feng, Huamei Chen, Jingjing Wu, Junyan Gao, Zheng Wen, Feng Huang, Ziwei Jiang

**Affiliations:** 1 The First School of Clinical Medicine, Guangzhou University of Chinese Medicine, NO. 12 Jichang Road, Baiyun District, Guangzhou, 510405 People’s Republic of China; 2 Clinical Medical College of Acupuncture, Moxibustion and Rehabilitation, Guangzhou University of Chinese Medicine, Guangzhou, 510006 People’s Republic of China; 3 Kunming Municipal Hospital of Traditional Chinese Medicine, The Third Affiliated Hospital of Yunnan University of Chinese Medicine, Kunming, 650011 People’s Republic of China; 4 Department of Orthopaedics & Traumatology, The First Affiliated Hospital of Guangzhou University of Chinese Medicine, NO. 16 Jichang Road, Baiyun District, Guangzhou, 510405 People’s Republic of China

**Keywords:** Bone defect, CiteSpace, Visual analysis, Web of Science, Bibliometrics

## Abstract

**Background:**

Bone defects can be seen everywhere in the clinic, but it is still a challenge for clinicians. Bibliometrics tool CiteSpace is based on the principle of “co-citation analysis theory” to reveal new technologies, hotspots, and trends in the medical field. In this study, CiteSpace was used to perform co-citation analysis on authors, countries (regions) and institutions, journals and cited journals, authors and cited literature, as well as keywords to reveal leaders, cooperative institutions, and research hotspots of bone defects and predict development trends.

**Method:**

Data related to bone defect from 1994 to 2019 were retrieved from the Web of Science core collection; then, we use Excel to construct an exponential function to predict the number of annual publications; conduct a descriptive analysis on the top 10 journals with the largest number of publications; and perform co-citation analysis on authors, countries (regions) and institutions, journals and cited journals, authors and cited reference, and keywords using CiteSpace V5.5 and use the Burst Detection Algorithm to perform analysis on the countries (regions) and institutions and keywords, as well as cluster the keywords using log-likelihood ratio.

**Results:**

A total of 5193 studies were retrieved, and the number of annual publications of bone defects showed an exponential function *Y* = 1×10^− 70e0.0829*x*^ (*R*^2^ = 0.9778). The high-yield author was Choi Seong-Ho at Yonsei University in South Korea. The high-yielding countries were the USA and Germany, and the high-yielding institutions were the Sao Paulo University and China and the Chinese Academy of Sciences which were the emerging research countries and institutions. The research results were mainly published in the fields of dentistry, bone, and metabolism. Among them, the Journal of Dental Research and Journal of Bone and Mineral Research were high-quality journals that report bone defect research, but the most cited journal was the Clinical Orthopaedics and Related Research. Hot keywords were regeneration, repair, in vitro, bone regeneration, reconstruction, and graft. The keywords that were strongly cited in 2010–2019 were transportation, osteogenic differentiation, proliferation, and biomaterials. After 2018, proliferation, osteogenic differentiation, stromal cells, transmission, and mechanical properties have become new vocabulary. The drug delivery, vascularization, osteogenic differentiation and biomaterial properties of bone defects were expected to be further studied.

**Conclusion:**

The application of CiteSpace can reveal the leaders, cooperating institutions and research hotspots of bone defects and provide references for new technologies and further research directions.

## Introduction

Bone defects caused by trauma, infection, and tumor can be seen everywhere in clinical practice. However, it is still a challenge for clinicians to promote the healing of bone defects [1]. Traditionally, free cancellous bone grafts have been repaired with varying degrees of bone resorption, even with blood supply or soft tissue coverage, resulting in delayed or nonunion [2]. Bone defects not only affect the quality of life of patients, but also brings a heavy economic burden and social burden [3]. Thus, it is very important to study how to accelerate the growth and healing of bone defects. However, with the changes of the times, new technologies, research hotspots, and development trends of bone defects are constantly changing. Therefore, it is of great significance to study the development trend and context of bone defects and explore new methods to promote the research and development of bone defects.

CiteSpace is a visual knowledge mapping bibliometrics tool invented by Chen Meichao of Drexel University based on Java language. It is mainly based on the principle of “co-citation analysis theory” and uses “pathfinder algorithm” to perform quantitative analysis on the literature in specific research fields and then presents the critical path of knowledge evolution in this field [4]. The concept of “co-citation analysis” was first proposed by American intelligence scientist Henry Small in 1973. When two documents appeared in the reference list of the third cited document, these two documents form a co-citation relationship. Co-citation analysis is the process of mining the co-citation relationship of a document spatial data collection [5]. It is generally believed that through the mining of the “co-citation relationship”, important knowledge turning points in related research fields can be revealed, and the analysis of the evolutionary potential dynamic mechanism of related research fields and the detection of frontiers in field development can be achieved [6]. CiteSpace has been widely used to study the hotspots and trends of diseases, providing a scientific basis for the prevention and treatment of diseases [7], such as spinal diseases [8] and traumatic diseases [9]. It quantifies the complex and diverse information through co-citation analysis and reveals the hidden meaning of the internal correlation and characteristics of the complex information [10]. It is undeniable that due to the high reliance on data, the analysis results may also be slightly different from the mainstream cognition in a field, resulting in pure quantitative analysis which may not be able to truly and accurately show the inner context of the development of the discipline. But the advantage of using quantitative analysis is also very obvious, that is, data analysis can be used to visually show the general development trend, network structure, and research hotspots in a field [11]. Therefore, the CiteSpace software can quantify the relationships between high-yield authors, countries and institutions, journals, and keywords, thus revealing new technologies, hotspots, and trends in the field of clinical medicine.

This study collects research data of bone defects in the Web of Science core collection and uses CiteSpace to perform co-citation analysis on authors, countries (regions) and institutions, journals and cited journals, authors and cited reference, and keywords, and combined with co-citation frequency, centrality, and related reference to explore new technologies, research hotspots, and trends of bone defects, which will provide a reference for in-depth exploration of this field. At the same time, it will promote cooperation between researchers, promote the application of new technologies, and solve difficult problems related to bone defects.

## Materials and methods

### Source

We used “bone defects” as the subject term or title to retrieve relevant research data from the Web of Science core collection from 1994 to 30 December 2019. The type of literature was not limited. The results were downloaded in plain text format of “Full Record and cited References”. At the same time, we also extracted information of the number of annual publications, research types, and the amount of publications of the journals.

### Descriptive analysis and fitting function construction

The number of annual publications, research types, and the amount of publications of the journals were imported into Excel 2013. The number of annual publications was calculated using Excel, and exponential functions, linear functions, logarithmic functions, and quadratic functions were used for trend representation, and high-fit functions were selected according to the magnitude of the correlation coefficient *R*^2^. The research types were classified and descriptively analyzed, and top 10 journals with the largest number of publications were screened, and their categories, journal ranking of Thomson Reuters, the impact factor (IF) of 2019, and the 5-year IF were analyzed.

### Co-citation analysis

Imported the data information of “Full records and cited references” into CiteSpace (V5.5 R2, invented by Chen Meichao of Drexel University). In terms of parameter settings, this study set the time span to 1994–2019, and time slice was set to one time partition every 1 year. Selected authors, countries (regions) and institutions, journals and cited journals, authors and cited reference, and keywords as the analysis objects for co-citation analysis, and choosed cosine as the connection strength. Screening criteria for authors, countries (regions) and institutions, journals and cited journals, authors and cited reference: Top was set to 5, which means that the top 5 data with the highest co-citation frequency or occurrences in each time segment were selected. The keyword selection criteria: Top was set to 10. The pathfinder algorithm was used to trim the co-cited map.

### Burst detection algorithm

In order to further screen out recent high-impact countries (regions), institutions, and hot keywords, we used the burst detection algorithm for analysis. In the parameter setting, this study set the time span to 1994–2019, and time slice was set to one time partition every 1 year. Used burst detection algorithms to analyze countries (regions) and institutions and keywords; choosed cosine as the connection strength. The screening criteria for countries (regions) and institutions: Top was set to 10, which means that the top 10 data with the highest co-citation frequency or occurrences in each time segment were selected. The screening criteria for keyword: Top was set to 15. The pathfinder algorithm was used to trim the co-cited map.

### Keyword clustering analysis

In order to distinguish the research situation of different topics, we used the log-likelihood ratio to cluster keywords. Since the keywords with higher positive likelihood ratio (LLR) are more representative of the characteristics of specific groups, we screened out the keywords with positive LLR ≥ 3.9 and *P* ≤ 0.05 in each group. The clustering results were analyzed by Timezone view to show the dynamic changes of time.

## Results and discussion

### Annual publications

**Fig. 1:**
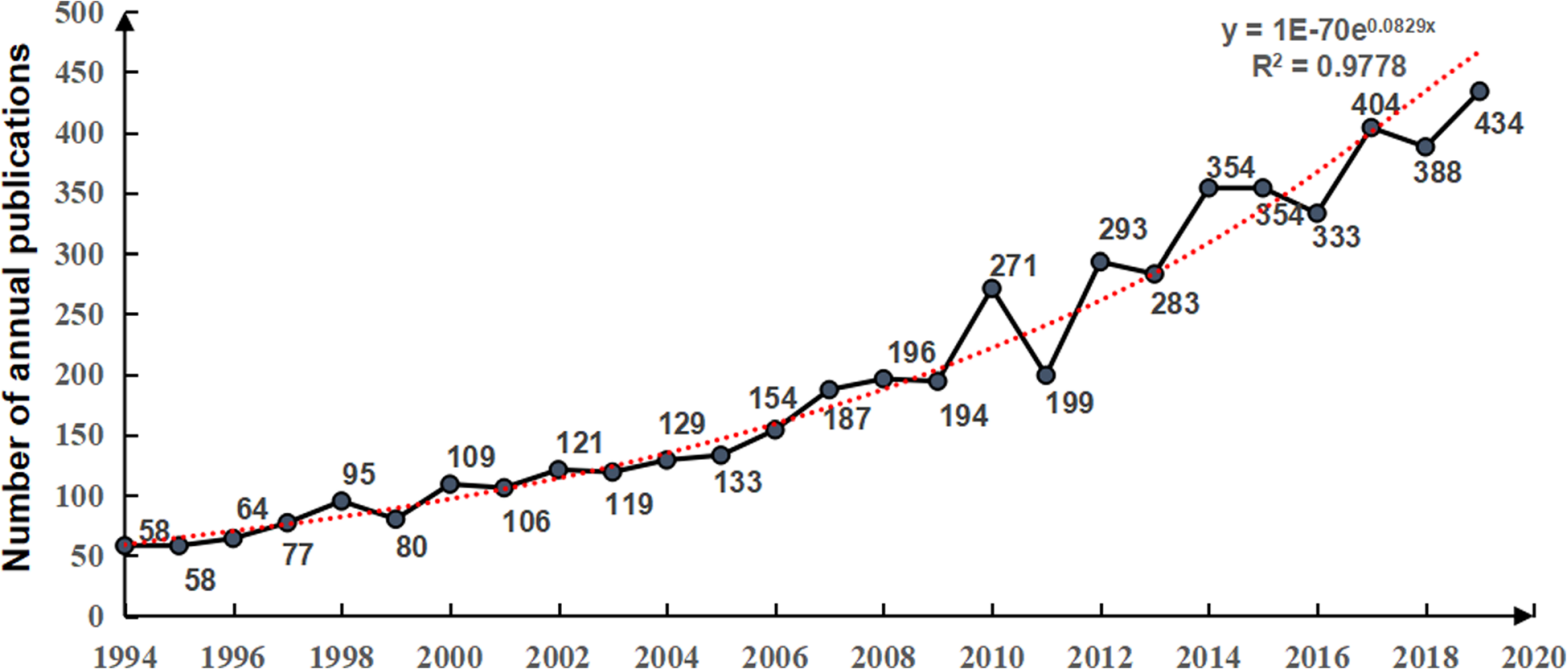
The number of annual publications

Figure 1 shows the annual publications of bone defects. From 1994 to 2010, the number of annual publications showed a trend of slow growth, while from 2011 to 2019, the number of annual publications showed a trend of increasing volatility. Among them, the number of annual publications in 2019 reached a peak, with a total of 434 articles. Previous studies have found that the introduction of induced membranes [12], the promotion of platelet-rich plasma technology [13], and the invention of biodegradable biomaterials [14] have contributed to the rapid development of bone defect studies after 2010. In order to further predict the change trend of bone defect studies, the index function *Y* = 1×10^−70e0.0829*x*^ (*R*^2^ = 0.9778, *Y* is the annual publications, *X* is the year) of the trend of annual publications of bone defects from 1994 to 2019 was screened according to the correlation coefficient *R*^2^, which reflected the rapid growth trend of bone defect studies in the past 25 years.

### Type of study

The types of studies included in this study are shown in Table 1. There are a total of 15 types of study, of which 4228 are research articles, accounting for 81.42%; 616 are meeting abstracts, accounting for 11.86%; and 128 are reviews, accounting for 2.46%. This suggests that most of the data came from original research. The most cited research article was published in *Nature* in 1996, with 1786 citations, which reported that B cell lymphogenesis and bone marrow myelogenesis were deficient in mice lacking the CXC chemokine PBSF/ SDF-1 [15]. The second highest cited research article was published in Arthroscopy-the Journal of Arthroscopic and Related Surgery in 2000, which reported traumatic glenohumeral bone defects and their relationship to failure of arthroscopic Bankart repairs [16]. The third highest cited research article was published in 1998 in Journal of Bone and Joint Surgery-American Volume, which reported the effect of autologous mesenchymal stem cell implants on the healing of canine segmental bone defects [17]. The most cited review article was published in Biomaterials in 2009, with 193 citations, which reported the challenge of establishing a pre-clinical model for segmental bone defect [18].

**Table 1:** Study types of the included data

No.	Study types	Amount	No.	Study types	Amount
1	Article	4228	9	Retracted publication	6
2	Meeting abstract	616	10	Retraction	6
3	Review	128	11	News item	3
4	Editorial material	105	12	Reprint	3
5	Letter	62	13	Book chapter	1
6	Correction	37	14	Correction addition	1
7	Early access	10	15	Discussion	1
8	Note	7			

### Author

**Fig. 2:**
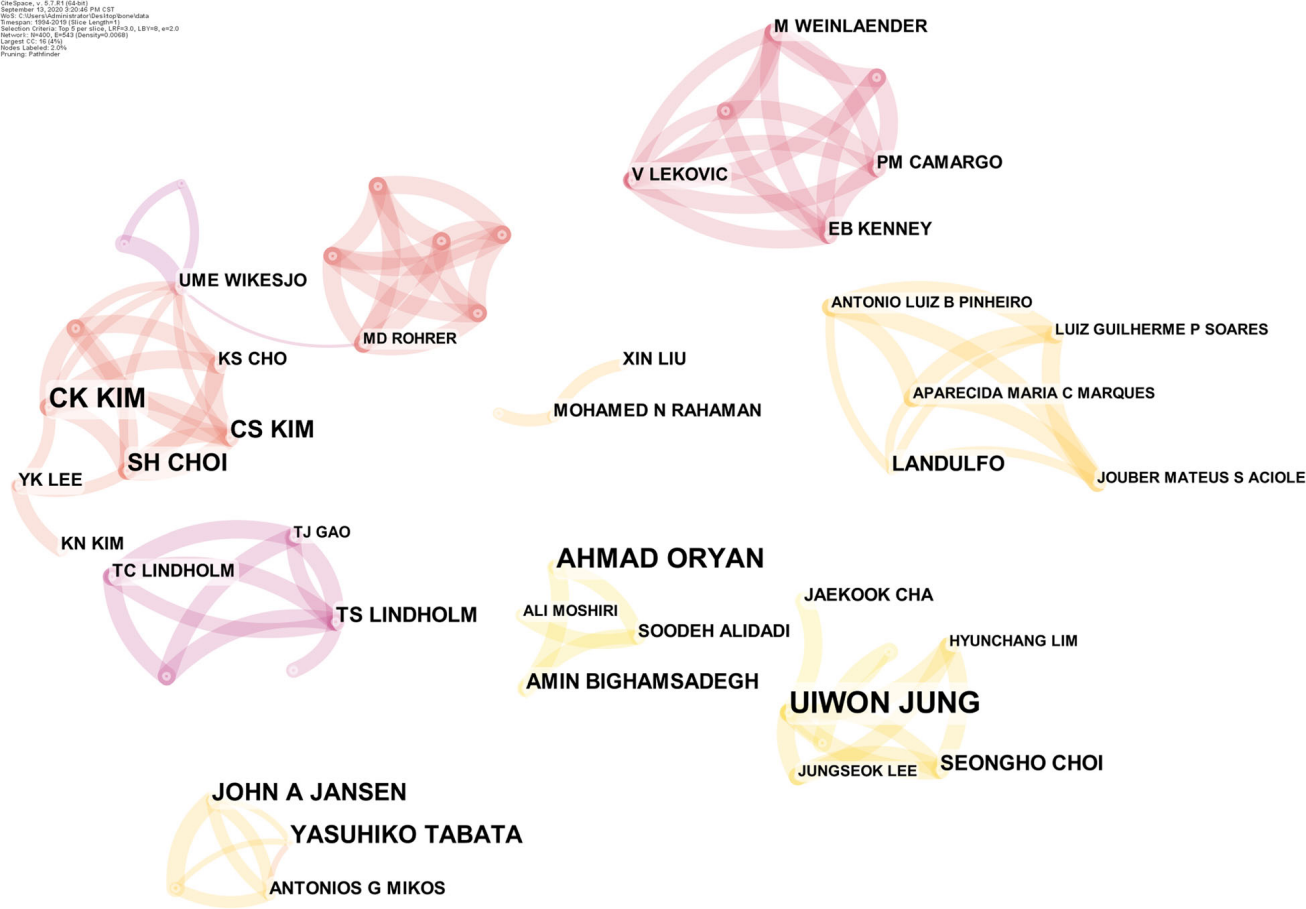
Map of author’s cooperative relationship. Note: *UIWON JUNG*, Jung Ui-Won; *CK KIM*, Kim Chong-Kwan; AHMAD ORYAN, Oryan Ahmad; *SH CHOI*, Choi Seong-Ho; *JOHN A JANSEN*, Jansen John A; *KS CHO*, CHO KS; *CS KIM*, Kim Chang-Sung; *YK LEE*, Lee Yong-Keun; *UME WIKESJO*, Wikesjoe Ulf M. E.

In order to discover high-impact authors in the field of bone defects, we used CiteSpace to analyze the co-citation of the authors, generating a distribution map of co-cited authors with 400 nodes and 543 connections (Fig. 2). According to the “co-citation analysis theory”, the large node represents the key node that dominates the entire co-cited network. The top 5 citations were Jung Ui-Won (13 times) and Kim Chong-Kwan (13 times) at Yonsei University in South Korea, Oryan Ahmad (13 times) at Shiraz University in Iran, Choi Seong-Ho (11 times) at Yonsei University in South Korea, and Jansen John A (11 times) at Nemegen University in the Netherlands. In terms of output quantity and cooperation, the high-yield author was Professor Choi Seong-Ho of Yonsei University in South Korea, focusing on the effects of tissue engineering materials on bone defect repair, such as 3D-printed polycaprolactone scaffold mixed with β-tricalcium phosphate as bone regeneration material for rabbit skull defect [19] and collagen membrane as a carrier of recombinant human bone morphogenetic protein 2 (RHBMP 2) to promote the regeneration of bone defects [20]. At the same time, it has established close cooperation with Kim Chong-Kwan, CHO KS, Kim Chang-Sung, Lee Yong-Keun, and Wikesjoe Ulf M. E [21, 22]..

### Countries (regions) and institutions

**Fig. 3:**
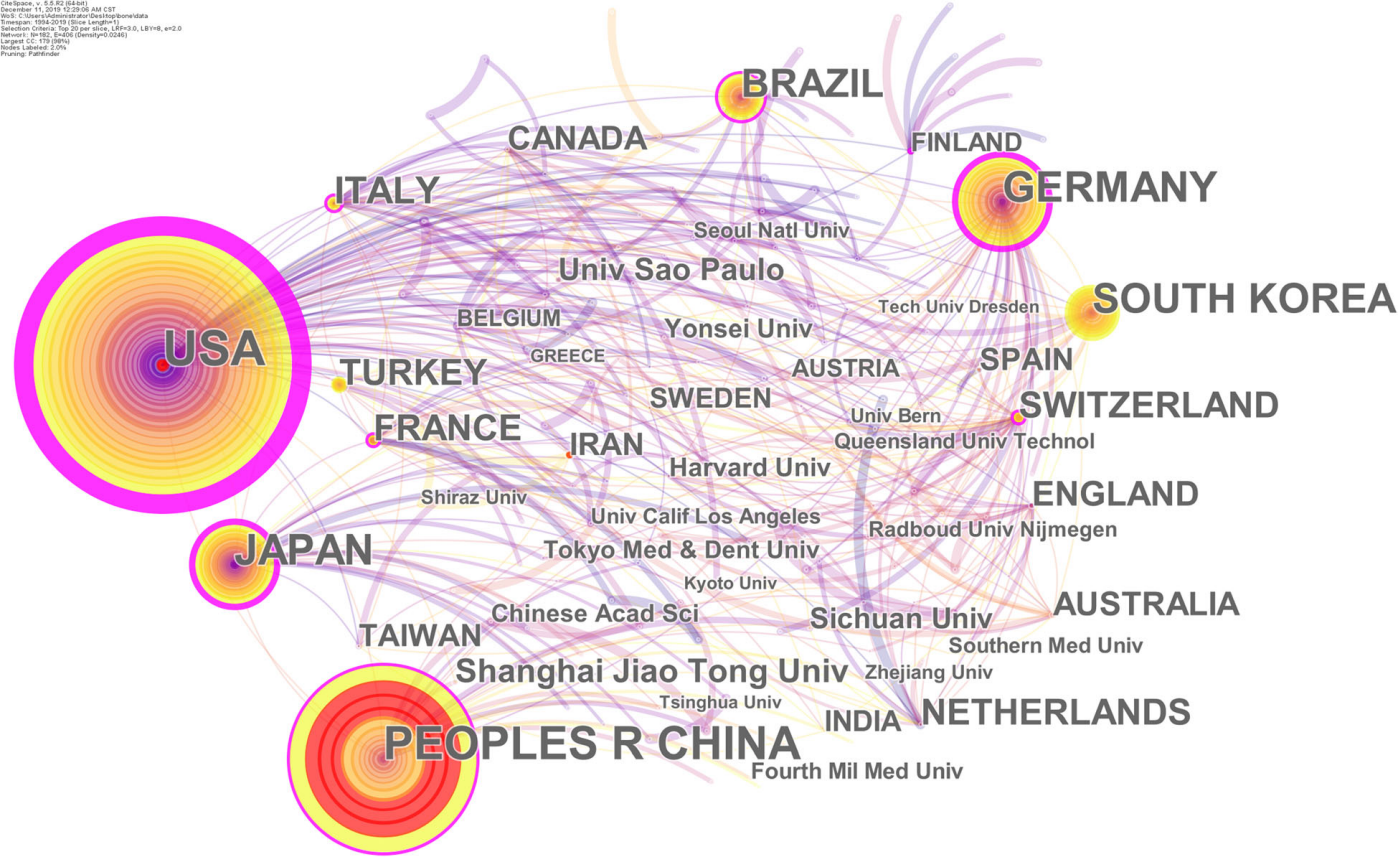
Map of countries (regions) and institutions’ cooperative relationship. Note: *Univ*, university; *PEOPLES R CHINA*, People’s Republic of China

In order to discover high-impact countries (regions) and institutions in the field of bone defects, we used CiteSpace to conduct a co-citation analysis of the countries (regions) and institutions. As shown in Fig. 3 and Supplementary Table 1, a total of 182 nodes and 406 connections in the countries (regions) and institutions co-citation network. The top 5 cited countries were the USA (1169 times), People’s Republic of China (824 times), Germany (480 times), Japan (452 times), and South Korea (319 times). The top 5 countries in centrality were the USA (1.1), Germany (0.26), Japan (0.2), Italy (0.17), and Finland (0.17). The top 5 cited institutions were Shanghai Jiaotong University (China, 93 times), Sao Paulo University (Brazil, 83 times), Sichuan University (China, 60 times), Yonsei University (South Korea, 41 times), and Harvard University (USA, 39 times). The top 5 institutions in centrality were Sao Paulo University (0.03), Harvard University (0.01), Radboud University Nijmegen (Netherlands, 0.01), Bernese University (Switzerland, 0.01), and University of Dusseldorf (Germany, 0.01). In terms of citation frequency and centrality, the most productive and cooperative countries (regions) were the USA, Germany, and Japan, while the institutions were Sao Paulo University and Harvard University. Cestari Tania Mary and Taga Rumio of the Sao Paulo University have published the most articles, with 12 articles each. They mainly study osteoinductive porous biphasic calcium phosphate ceramics as an alternative to autogenous bone transplantating for the treatment of mandibular bone critical-size defects [23], the effect and mechanism of sintered anorganic bone graft in repairing bone defects [24, 25]. Evans CH and Glimcher MJ have the most published articles at Harvard University, all reaching seven articles. They mainly study how to quickly and reliably cure severe bone defects through genetic modification [26], and the osteogenic characteristics of decalcified bone matrix induced rat skull defects [27]. The USA has cooperation with China, Germany, Japan, Brazil, South Korea, etc., while Germany, Japan, and Sweden have the closest cooperation relationships. The Sao Paulo University cooperates closely with Universidade Federal da Bahia [25] and the University of Texas System [28]. Harvard University works closely with Nemegen University [29] and Queensland University of Technology (Australia) [30].

**Fig. 4:**
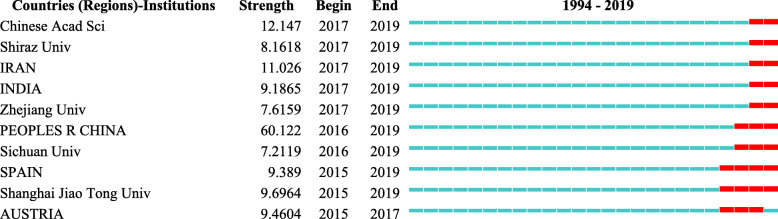
Top 10 countries (regions) and institutions with the strongest citation bursts. Note: *Chinese Acad of Sci*, Chinese Academy of Sciences; *Univ*, university; *PEOPLES R CHINA*, People’s Republic of China

In order to further explore the recent high-impact countries (regions) and institutions, we use the burst detection algorithm to analyze the countries (regions) and institutions, which can get the countries (regions) and/or institutions that have attracted the close attention of the academic community, and with large citations in a certain period of time. Countries (regions) and/or institutions with high burst values mean that their research output and influence are high in the corresponding time period. The top 10 countries (regions) and institutions in burst values are shown in Fig. 4: the countries (regions) are Iran (11.03), India (9.19), China (60.12), Spain (9.39), and Austria (9.46), while the institutions are Chinese Academy of Sciences (12.15), Shiraz University (8.16), Zhejiang University (7.62), Sichuan University (7.21), and Shanghai Jiaotong University (9.70). China has become an emerging country in the field of bone defects with the highest burst value of about 60.12, which appeared during 2016–2019. In terms of the number of citations, in 2016, Shanghai Jiaotong University and Fudan University found that exosomes secreted by human-induced pluripotent stem cell-derived mesenchymal stem cells repair critical-size bone defects by enhancing angiogenesis and osteogenesis, which has received widespread attention [31]. In 2017, the study of bone grafts and biomaterial substitutes systematically summarized by Wang WH from the University of Hong Kong was strongly cited [32]. The burst detection algorithm analysis of institutional found that the Chinese Academy of Sciences has become the most cited institution, and its studies were strongly cited in 2017–2019, with a burst value of approximately 12.147. The high-yield author of the Chinese Academy of Sciences is Qin L, who has published eight studies focusing on the performance and effectiveness of PLGA/TCP porous scaffolds in repairing bone defects [33, 34].

### Journals and cited journals

The 5193 articles retrieved in this study were published in 100 journals, with an average of 51.93 articles in each journal. The field of distribution is relatively scattered, reflecting that the studies on the topic of bone defects involves a wide range of subjects and has obvious interdisciplinary characteristics. The top 10 journals in terms of publications were Tissue Engineering Part A (140), Journal of Craniofacial Surgery (136), Journal of Periodontology (122), Bone (119), Journal of Dental Research (111), Journal of Bone and Mineral Research (105), Clinical Oral Implants Research (103), Journal of Biomedical Materials Research Part A (88), Biomaterials (86), and Journal of Biomedical Materials Research Part B Applied Biomaterials (79), as shown in Table 2. The total number of articles published by these 10 journals is 1089, accounting for 20.97%. Besides, the studies of bone defects were mainly published in the following eight fields: biotechnology and applied microbiology, cell and tissue engineering, cell biology, surgery, dentistry, endocrinology and metabolism, engineering, and materials science. From the perspective of influence, the average IF of these 10 journals in 2019 was 4.351 points, 7/10 journals “2019 IF” > 3 points, 8/10 journals “5-year IF” > 3 points, 5/10 journals were ranked first in the Thomson Reuters journal rankings. In terms of publications, Journal Ranking and IF, Journal of Dental Research, and Journal of Bone and Mineral Research are high-quality journals that report bone defects. Journal of Dental Research mainly reports on clinical research, biomaterials, and bioengineering of dentistry, while Journal of Bone and Mineral Research mainly reports on bone and metabolism related studies, such as bone, bone and skeletal system, and mineral metabolism.

**Table 2:** Top 10 journals with the largest number of publications

No.	Journal	Number of publications	Category (ranking)	Journal ranking of Thomson Reuters	2019IF	5-year IF
1	Tissue Engineering Part A*	140	Biotechnology & Applied Microbiology (44/161); Cell & Tissue Engineering (13/24); Cell Biology (89/190)	Q2	3.508	4.145
2	Journal of Craniofacial Surgery	136	Surgery (183/210)	Q4	0.953	1.05
3	Journal of Periodontology	122	Dentistry, Oral Surgery & Medicine (7/91)	Q1	3.742	3.614
4	Bone	119	Endocrinology & Metabolism (36/143)	Q2	4.147	4.349
5	Journal of Dental Research	111	Dentistry, Oral Surgery & Medicine (3/91)	Q1	4.914	5.844
6	Journal of Bone and Mineral Research	105	Endocrinology & Metabolism (19/143)	Q1	5.854	5.985
7	Clinical Oral Implants Research	103	Engineering, Biomedical (21/87) Dentistry, Oral Surgery & Medicine (8/91)	Q1	3.723	4.044
8	Journal Of Biomedical Materials Research Part A	88	Materials Science, Biomaterials (16/38) Engineering, Biomedical (23/87)	Q2	3.525	3.469
9	Biomaterials	86	Engineering, Biomedical (4/87) Materials Science, Biomaterials (1/38)	Q1	10.317	9.656
10	Journal Of Biomedical Materials Research Part B Applied Biomaterials	79	Engineering, Biomedical (35/87) Materials Science, Biomaterials (23/38)	Q2	2.831	2.882

*Tissue Engineering Part A only have 2017IF

**Fig. 5:**
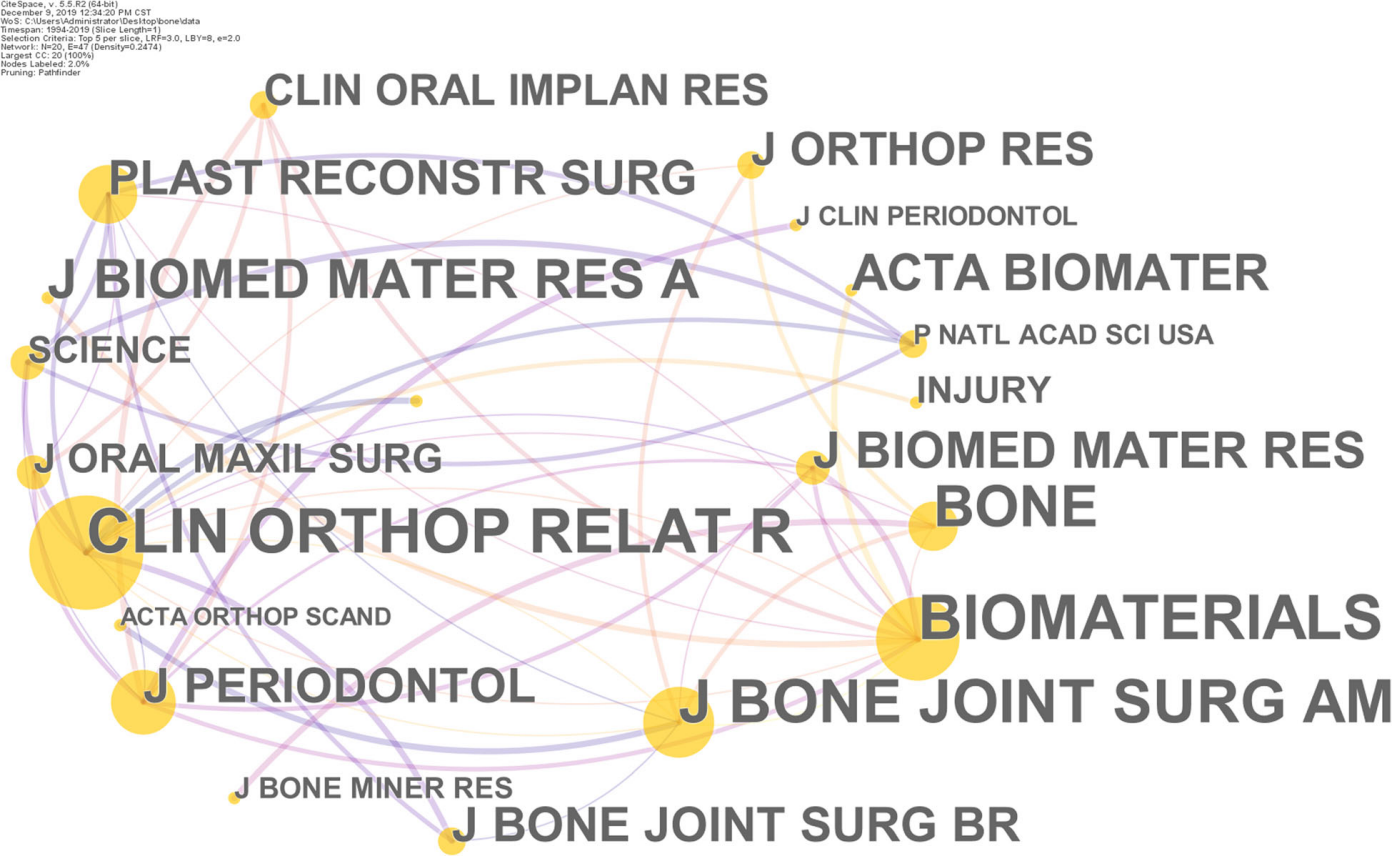
Map of journal co-citation. Note: *Clin Orthop Relat R*, Clinical Orthopaedics and Related Research; *J Bone Joint Surg AM*, Journal of Bone and Joint Surgery-American Volume; *J Biomed Mater Res A*, Journal of Biomedical Materials Research Part A; *Clin Orthop Relat R*, Clinical Orthopaedics and Related Research; *J Periodontol*, Journal of Periodontology

Journals with high publication capacity may not be the mainstream journals in specific research fields, but the distribution of journals that are of concern to academia can be obtained through the analysis of “co-cited journals”. The co-citation frequency reflects the quality and influence of the journal. Journals with high co-citation frequency are often regarded by academic circles as mainstream journals. The co-citation analysis of the cited journals obtained from CiteSpace was shown in Fig. 5 and Supplementary Table 2, involving 20 nodes and 47 connections. The larger the node, the higher the co-citation frequency of the journal. The top 5 journals in terms of co-citation frequency were Clinical Orthopaedics and Related Research (2091 times), Biomaterials (1845 times), Journal of Bone and Joint Surgery-American Volume (1806 times), Bone (1095 times), and Journal of Biomedical Materials Research Part A (781 times). The top 5 journals in terms of centrality were Clinical Orthopaedics and Related Research (0.84), Biomaterials (0.56), Journal of Bone and Joint Surgery-American Volume (0.29), Journal of Periodontology (0.28), and Bone (0.22). Obviously, there is no complete correspondence between the top co-cited journals and the journals with the high publications, and some journals with the high publications have not attracted the common attention of the academic community. According to the co-citation frequency and centrality, the Clinical Orthopaedics and Related Research is the current mainstream journal, which belongs to Q1 of Orthopedics and Surgery field. The 2019IF of Clinical Orthopaedics and Related Research is 4.329, and the annual publications are about 500. It mainly focuses on the diagnosis and treatment of musculoskeletal, and its published research can focus on the research basis of bone defects.

### Author and cited reference

**Fig. 6:**
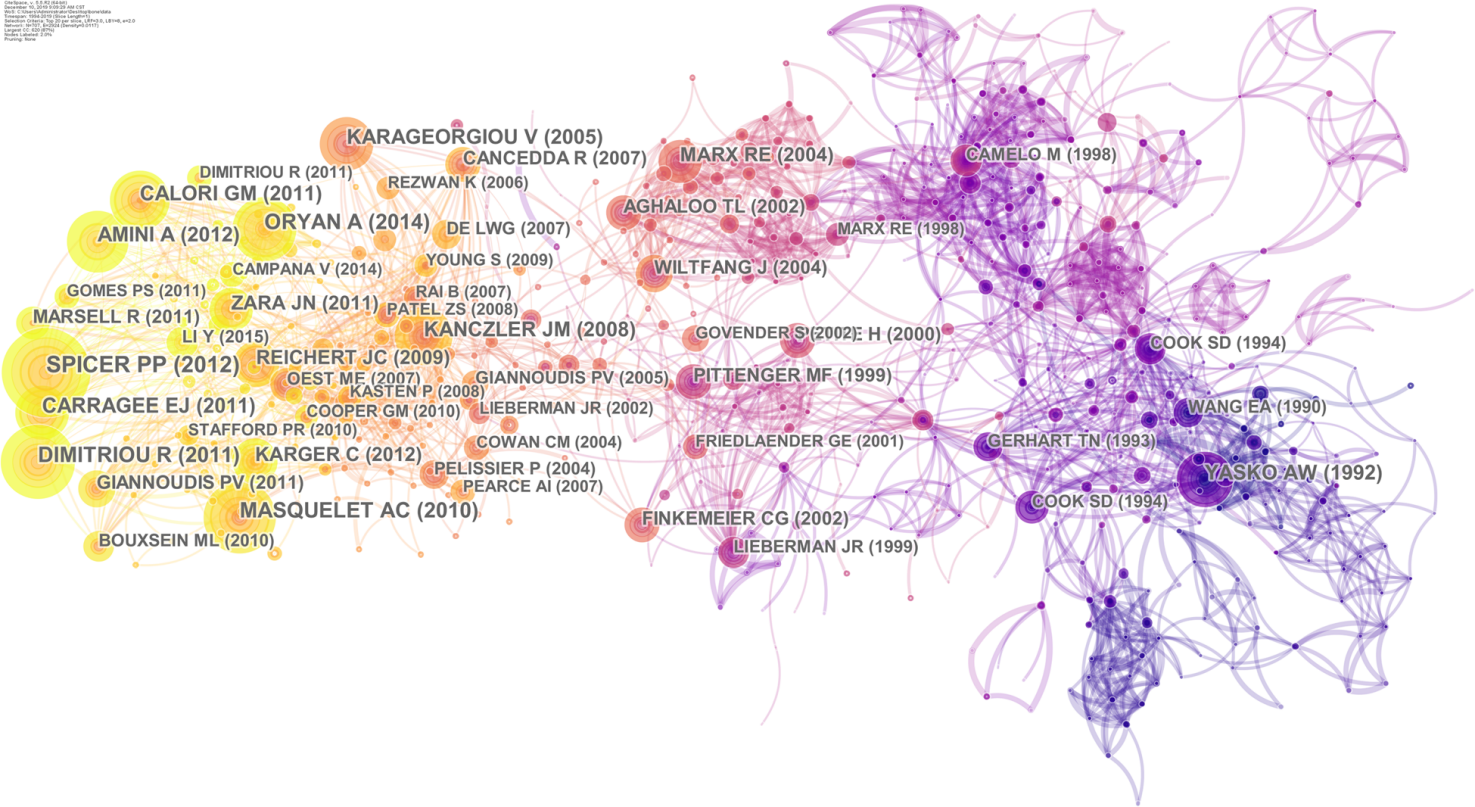
Map of author and reference co-citation

In the references’ co-citation networks generated by CiteSpace, key reference nodes refer to nodes connecting two or more clusters. The key references have a high co-citation frequency, occupy a key position in the knowledge flow network, and are the basis of knowledge research in a discipline. The co-citation network of the author and the cited reference is shown in Fig. 6, with 707 nodes and 2924 connections. It mainly involves two original articles and three review articles (Table 3). The contents involved are the evaluation of bone regeneration using critical-size calvarial defects of rats [35]; the concept of induced membrane for reconstruction of long bone defects [12]; bone regeneration: current concepts and future directions [36]; bone regenerative medicine: classic choices, novel strategies, and future directions [37]; and osteogenesis and angiogenesis: the potential of bone engineering [38]. Obviously, these key references are the basis of bone defects research.

**Table 3:** Top 5 author and reference co-citation

No.	Frequency	Study types	Cited reference	Author (year of publication)
1	57	Article	Evaluation of bone regeneration using the rat critical size calvarial defect	SPICER PP (2012) [35]
2	47	Article	The concept of induced membrane for reconstruction of long bone defects	MASQUELET AC (2010) [12]
3	47	Review	Bone regeneration: current concepts and future directions	DIMITRIOU R (2011) [36]
4	41	Review	Bone regenerative medicine: classic options, novel strategies, and future directions	ORYAN A (2014) [37]
5	40	Review	Osteogenesis and angiogenesis: the potential for engineering bone	KANCZLER JM (2008) [38]

### Analysis of keyword co-citation, burst value, clustering and time evolution

**Fig. 7:**
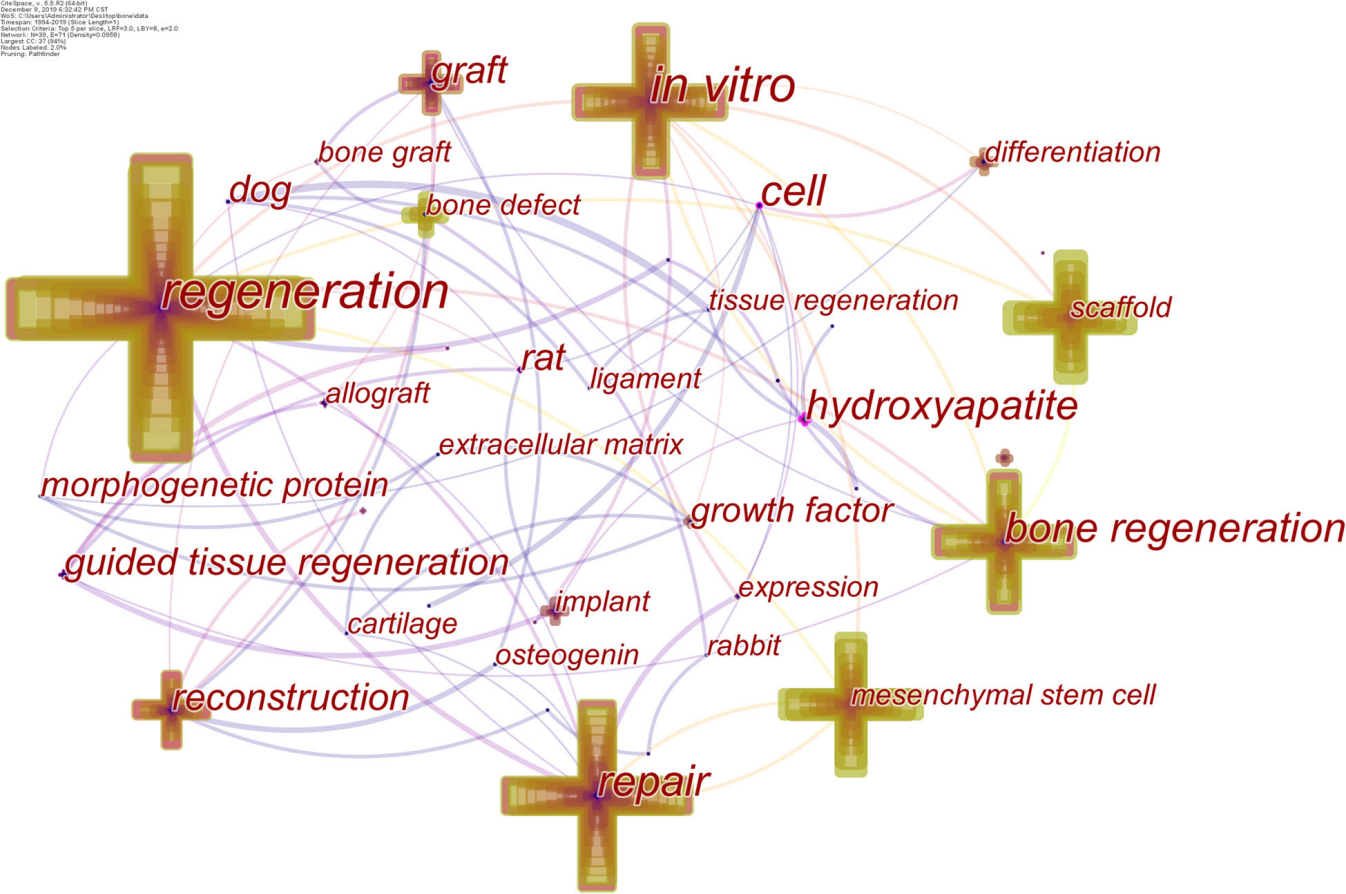
Map of keywords co-citation

Highly cited keywords reflect the focus of attention of a subject and are the direction of continuous development of a subject. We perform co-citation analysis of keywords to identify hot topics and urgent problems in the field of bone defects. The keyword co-citation network generated by CiteSpace was shown in Fig. 7 and Supplementary Table 3, with 39 nodes and 71 connections. The top 10 keywords in co-citation frequency are regeneration (757 times), repair (508 times), in vitro (381 times), bone regeneration (360 times), mesenchymal stem cells (347 times), scaffold (306 times), reconstruction (233 times), graft (156 times), bone defect (107 times), and implant (105 times). The top 10 keywords in centrality are regeneration (0.44), in vitro (0.39), cell (0.35), repair (0.29), hydroxyapatite (0.26), bone regeneration (0.25), reconstruction (0.20), graft (0.16), guided tissue regeneration (0.14), and rabbit (0.14). In terms of co-citation frequency and centrality, regeneration, repair, in vitro, bone regeneration, reconstruction, and graft are hot keywords, which indicated that in the in vitro study, bone regeneration and reconstruction, as well as bone transplantation, have received wide attention.

**Fig. 8:**
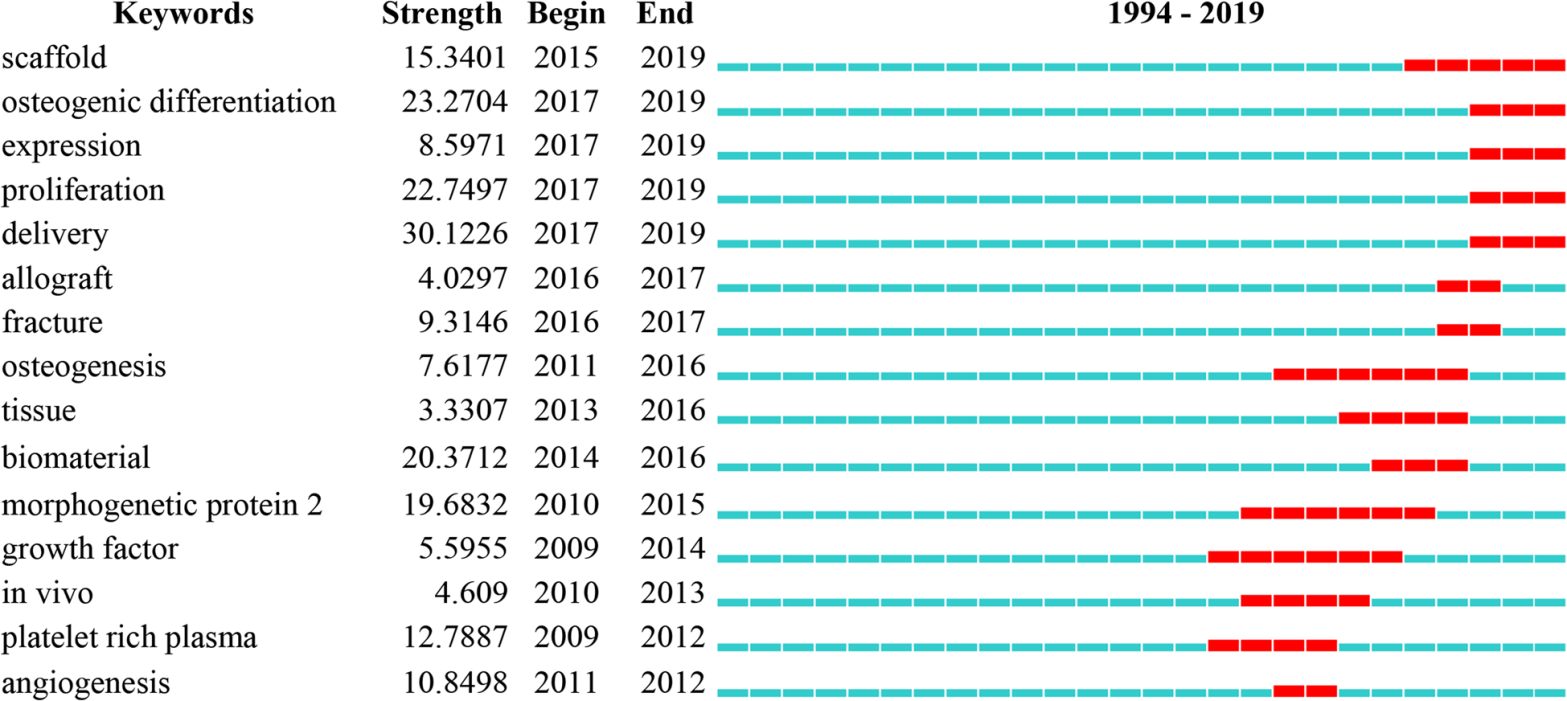
Top 15 keywords in the burst value from 2010 to 2019

In order to obtain keywords that have recently attracted close attention from the academic community, we further use the burst detection algorithm to analyze the keywords. This analysis method can display the results in two dimensions of the burst value and the burst time. The keywords with high burst value in a period of time mean that they have received special attention in the corresponding time interval, and to some extent represent the research frontier of the research field in the corresponding time interval. The top 15 keywords in the burst value are shown in Fig. 8, and the red nodes represent the annual rings. During 2010–2019, delivery, osteogenic differentiation, proliferation, and biomaterial were strongly cited, with the burst values of 30.12, 23.27, 22.75, and 20.37, respectively, and most of them occurred during the period of 2017–2019. This indicates that biomaterials play a role in drug delivery, promoting bone proliferation and differentiation in the repair of bone defects, which may be a research hotspot in recent bone defects.

The maximum likelihood estimation method was first proposed by the German mathematician CF Gauss in 1821. RA Fisher further explored the nature of this method in 1922, which is currently commonly used in cluster analysis to distinguish the characteristics of different things. Clustering analysis of keywords using log-likelihood ratio can clearly show the internal characteristics of the research object and provide reliable evidence for predicting the evolution of research hotspots. Keywords with a larger LLR are more representative of this cluster. We set the positive LLR ≥ 3.9 and *P* ≤ 0.05 as the screening conditions to screen the keywords. Keywords are divided into seven categories, as shown in Table 4. The research hotspots of bone defects are distributed as follows: management of bone transplantation, dental bone regeneration technology, bone formation in bone defect rats, the application of bone tissue engineering such as biological scaffolds in the vascularization and osteogenic differentiation of bone defects, membrane-induced bone regeneration, nanomaterials induce bone formation, and extracellular matrix induces cartilage formation and differentiation.

**Table 4:** Cluster analysis of keywords

Cluster ID	Keywords (positive likelihood ratio, P)	Content
1	management (11.22, 0.001); bone graft (8.98, 0.005); reconstruction (8.98, 0.005); bone regeneration (7.43, 0.01); allograft (6.78, 0.01); rat (6.2, 0.05); in vitro (5.63, 0.05); impaction bone grafting (5.6, 0.05); augmentation (5.6, 0.05); transport (5.6, 0.05); vascularized fibular graft (5.6, 0.05); children (5.6, 0.05); lumbar hernia (5.6, 0.05); dental implant (5.6, 0.05); bone tissue engineering (4.49, 0.05); periodontal regeneration (4.49, 0.05); calvarial defect (4.49, 0.05); marrow (4.47, 0.05); model (4.47, 0.05); guided tissue regeneration (4.31, 0.05); bone defect (3.91, 0.05)	Management of bone graft
2	bone regeneration (17.05, 1.0E-4); periodontal regeneration (13.4, 0.001); mandibular molar (10.81, 0.005); guided tissue regeneration (8.64, 0.005); freeze dried bone (7.2, 0.01); clinical evaluation (7.2, 0.01); alveolar bone fill (7.2, 0.01); radiography (7.2, 0.01); reconstructive osseous surgery (7.2, 0.01); membranes artificial (7.2, 0.01); intrabony defects (7.2, 0.01); osseous grafting (7.2, 0.01); peri-implantitis (6.67, 0.01)	Dental bone regeneration technology
3	rat (15.58, 1.0E-4); demineralized bone (14.88, 0.001); purification (10.55, 0.005); induction (9.9, 0.005); composite (9.9, 0.005); osteogenin (9.9, 0.005); bone freeze-dried (6.26, 0.05); porous hydroxyapatite (6.26, 0.05); inductive protein (6.26, 0.05); rabbit (6.01, 0.05); osteoblast (4.94, 0.05); matrix (4.94, 0.05); osteogenic effect (4.94, 0.05); periradicular surgery (4.94, 0.05); fibrin collagen paste (4.94, 0.05); decalcified bone (4.94, 0.05); hormone (4.94, 0.05); bone/demineralized (4.94, 0.05); nasal tip (4.94, 0.05); local application (4.94, 0.05); periodontitis therapy (4.94, 0.05); male animal (4.94, 0.05); e-ptfe (4.94, 0.05); parietal bone (4.94, 0.05); gentamicin (4.94, 0.05); bioerodible polyorthoester (4.94, 0.05); tetracycline therapeutic use (4.94, 0.05); mesenchymal cell (4.94, 0.05); artificial membranes (4.94, 0.05); skull (4.94, 0.05); gastric pentadecapeptide bpc-157 (4.94, 0.05); doxycycline (4.94, 0.05); family member (4.94, 0.05); skull surgery (4.94, 0.05); periodontal pockets therapy (4.94, 0.05); periodontal diseases therapy (4.94, 0.05); segmental bone defects (4.94, 0.05); calvarial bone defects (4.94, 0.05); experimental (4.94, 0.05); autologous cortical bone (4.94, 0.05); polytetrafluoroethylene (therapeutic use) (4.94, 0.05); octacalcium phosphate (4.94, 0.05); substitute (4.94, 0.05); capacity (4.94, 0.05); intramuscular application (4.94, 0.05); stomach (4.94, 0.05); prostaglandin (4.94, 0.05); male rats (4.94, 0.05); attachment (4.94, 0.05); morphogenetic protein (4.71, 0.05); implantation (4.71, 0.05)	Bone formation in rats with bone defects
4	bone tissue engineering (19.2, 1.0E-4); angiogenesis (14.38, 0.001); guided tissue regeneration (12.29, 0.001); calvarial defect (11.47, 0.001); membranes (10.82, 0.005); osteogenic differentiation (9.57, 0.005); mesenchymal stem cell (6.81, 0.01); in vitro (6.71, 0.01); bone graft (5.71, 0.05); reconstruction (5.71, 0.05); periodontal diseases/therapy (5.71, 0.05); demineralized bone matrix (5.29, 0.05); scaffold (5.07, 0.05); hydroxyapatite/therapeutic use (4.99, 0.05); polycaprolactone (4.78, 0.05); beta tricalcium phosphate (4.78, 0.05); marrow stromal cell (4.78, 0.05); vivo (4.78, 0.05); femoral defect (4.78, 0.05); articular cartilage (4.78, 0.05); vitro (4.78, 0.05); proliferation (4.78, 0.05); bmscs (4.78, 0.05); hydroxyapatite scaffold (4.78, 0.05); mandibular reconstruction (4.78, 0.05); delivery (4.78, 0.05); guided bone regeneration (4.61, 0.05); bone substitutes (4.26, 0.05); periodontal diseases/surgery (4.26, 0.05); barrier (4.26, 0.05); outcome assessment (4.26, 0.05)	Application of bone tissue engineering such as biological scaffolds in vascularization and osteogenic differentiation of bone defects
5	membranes (21.65, 1.0E-4); guided bone regeneration (12.99, 0.001); bioabsorbable (11.02, 0.001); comparison studies (11.02, 0.001); barrier (7.52, 0.01); mandible (6.56, 0.05); grafts bone (6.56, 0.05); periodontal diseases/therapy (5.57, 0.05); implant-associated defect (5.1, 0.05); component (5.1, 0.05); cell binding peptide (5.1, 0.05); oral implants (5.1, 0.05); bone allografts (5.1, 0.05); membranes bioabsorbable (5.1, 0.05); gingival recession/therapy (5.1, 0.05); freeze-dried bone (5.1, 0.05); defect (5.1, 0.05); calcium sulfate/therapeutic use (5.1, 0.05); intraosseous defects (5.1, 0.05); gap bone filler (5.1, 0.05); gingival recession/surgery (5.1, 0.05); follow-up studies (5.1, 0.05); gtam (5.1, 0.05); gene expression (5.1, 0.05); controlled (5.1, 0.05); collagen synthesis (5.1, 0.05); laminar bone (5.1, 0.05); dogs (5.1, 0.05); graft healing (5.1, 0.05); implants (5.1, 0.05); osseointegration (5.1, 0.05); acetabulum (5.1, 0.05); osteocalcin (5.1, 0.05); lllt (5.1, 0.05); polytetrafluoroethylene/therapeutic use (5.1, 0.05); alveolar bone loss (5.1, 0.05); resorbable membrane (5.1, 0.05); graft shape (5.1, 0.05); mta (5.1, 0.05); lyodura (5.1, 0.05); finite element analysis (5.1, 0.05); grafts (5, 0.05); bone (4.86, 0.05); guided tissue regeneration (4.02, 0.05); autograft (3.97, 0.05); cancellous bone (3.97, 0.05); clinical trials (3.97, 0.05); artificial (3.97, 0.05); repair (3.76, 0.1)	Membrane-induced bone regeneration
6	hydroxyapatite (14.12, 0.001); osteoconduction (10.26, 0.005); mechanical properties (10.26, 0.005); mechanical property (6.6, 0.05); carbon nanotube (5.12, 0.05); bioactive glas (5.12, 0.05); extrusion free-forming (5.12, 0.05); osteoinduction (5.12, 0.05); arthroplasty (5.12, 0.05); haemocompatibility (5.12, 0.05); repetitive acidic amino acid (5.12, 0.05); composite and powder materials (5.12, 0.05); nanocrystalline hydroxyapatite (5.12, 0.05); bone morphogenic protein 2-related peptide (5.12, 0.05); collagen scaffold (5.12, 0.05); microsphere (5.12, 0.05); nanowire (5.12, 0.05); bone cement (5.12, 0.05); porous tantalum (5.12, 0.05); tetracycline (5.12, 0.05); production process (5.12, 0.05); structure (5.12, 0.05); carbon implants (5.12, 0.05); led phototherapy (5.12, 0.05); nano-hydroxyapatite (5.12, 0.05); bone-implant contact (5.12, 0.05); cement (5.12, 0.05); strength (5.12, 0.05); bioglass (5.12, 0.05); cross linking (5.12, 0.05); nanostructured materials (5.12, 0.05); normal bone (5.12, 0.05); tgf-beta 1 (5.12, 0.05); membrane technique (5.12, 0.05); sintering temperature (5.12, 0.05); silk fibroin (5.12, 0.05); bioactive glass (5.12, 0.05); pattern (5.12, 0.05); slurry-compounding process (5.12, 0.05); drug delivery (5.12, 0.05); new bone formation (5.12, 0.05); hip (5.12, 0.05); weight bearing fracture (5.12, 0.05); early vascularization (5.12, 0.05); lactosorb (5.12, 0.05); tibial fracture (5.03, 0.05); biomaterials (4.01, 0.05); biomaterial (4.01, 0.05)	Nanomaterials induce bone formation
7	extracellular matrix (14.6, 0.001); cartilage (12.45, 0.001); bovine bone (8.91, 0.005); collagen (8.91, 0.005); beta family (8.91, 0.005); mandibular defects (6.3, 0.05); smooth muscle cell (6.3, 0.05); osteogenic protein (6.3, 0.05); camurati engelmann disease (6.3, 0.05); human patients (6.3, 0.05); ectopic induction (6.3, 0.05); transforming growth factor beta 1 (6.3, 0.05); water soluble protein (6.3, 0.05); plate (6.3, 0.05); member (6.3, 0.05); bone induction (6.3, 0.05); colony-forming unit fibroblastic (6.3, 0.05); increased expression (6.3, 0.05); mandibular fracture (6.3, 0.05); cartilage differentiation (6.3, 0.05); hydroxyapatite granules (6.3, 0.05); delivery vehicle (6.3, 0.05); biglycan (6.3, 0.05); osteoinductive implants (6.3, 0.05); fibroblast cells (6.3, 0.05); screw (6.3, 0.05); indian hedgehog (6.3, 0.05); 8 cysteine repeat (6.3, 0.05); new bone (6.3, 0.05); fibronectin (6.3, 0.05)	Extracellular matrix induces cartilage formation and differentiation

**Fig. 9:**
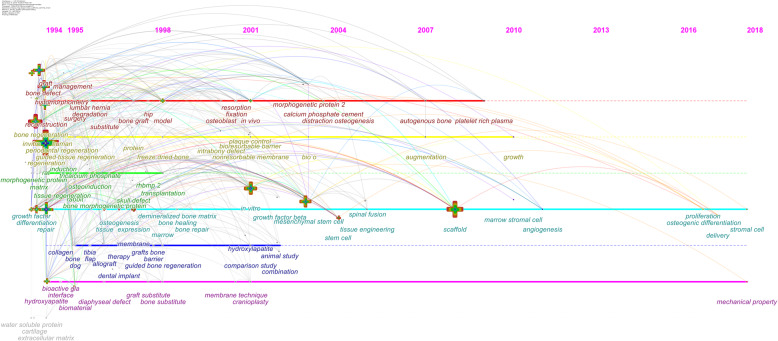
Timezone view of keywords. (The node indicates that the keyword appears for the first time in large numbers, and the arc indicates that it is cited)

In order to further explore the time evolution characteristics of different clusters, we performed Timezone view analysis to show the dynamic changes of different cluster keywords, as shown in Fig. 9. The time evolution of clusters is as follows:

1. In terms of “the management of bone transplantation”, it received wide attention from 1994 to 2009. In detail, in 1994, bone transplantation, management, bone defect, histomorphometrical, reconstruction, and degradation were hot keywords [39]. In 2001, in vivo, resorption, fixation, and osteoblast became a new research direction [40]. In 2003, calcium phosphate cement, morphogenetic protein 2, distraction, and osteogenesis became hot keywords, indicating that the promotion of osteogenesis by calcium phosphate cement was a new focus in this period [41]. In 2007, the application of autogenous bone platelet-rich plasma in bone defects became a hot trend [42].
2. In terms of “dental bone regeneration technology”, it received attention from 1994 to 2010. In detail, in 1994, bone regeneration, in vitro, periodontal regeneration, guided tissue regeneration, and regeneration have became hot keywords, indicating that tooth regeneration technology has received widespread attention at this stage [43]. In 1998, freeze-dried bone transplantation became a new focus [44]. In 2000, plaque control, bioresorbable barrier, intrabony defect, and nonresorbable membrane became new research directions [45]. From 2007 to 2010, augmentation and growth were hot keywords, indicating that augmentation of bone proliferation was gaining widespread attention [46].
3. In terms of “bone formation in bone defect rats”, it has been received wide attention from 1994 to 1998. In detail, in 1994, morphogenetic protein, induction, tissue regeneration, bone morphogenetic protein, and tricalcium phosphate have became hot words, which means that the promotion of bone defect formation by tricalcium phosphate has received wide attention during this period. In 1998, RHBMP 2 and transplantation became research hotspot. Even in 2008, when the biological scaffold was vigorously applied, it still received attention.
4. In terms of “the application of bone tissue engineering such as biological scaffolds in the vascularization and osteogenic differentiation of bone defects”, it has been a research hotspot since 1994. In detail, in 1994, growth factor, differentiation, and repair were hot keywords. In 1998, osteogenesis, tissue expression, demineralized bone matrix, bone healing, bone repair, and marrow became hot terms. In 2004, tissue engineering and stem cells became new hotspots. In 2010, marrow stromal cells and angiogenesis became new research trends. In 2010, marrow stromal cells, angiogenesis, and other blood vessels related to bone repair issues become hot research subjects. In 2017, proliferation, osteogenic differentiation, stromal cells, and delivery were hot keywords, suggesting that drug delivery, vascularization, and osteogenic differentiation of bone defects became new research directions.
5. In terms of “membrane-induced bone regeneration”, it received attention from 1995 to 2002. In detail, in 1995, collagen, tibia, and allograft were hot keywords. In 1998, membrane, grafts bone, barrier, guided bone regeneration, and dental implant were hot keywords, indicating that the role of membrane in bone defect restoration has attracted attention [47]. In 2001, hydroxyapatite, comparison study, and animal study were hot keywords, indicating that hydroxyapatite repairing bone defects became a hot research in this period.
6. The theme of “nanomaterials induce bone formation” has been a research hotspot since 1994. In detail, in 1994, bioactive gla, interface, biomaterial, and diaphyseal defect were hot keywords. In 1998, graft sbstitute and bone substitute became a new hotspot. In 2001, the application of membrane technology in bone defect repair and cranioplasty received in-depth research. In 2018, the mechanical properties of new materials have became a research hotspot [48].
7. “Extracellular matrix induces cartilage formation and differentiation” began to appear in 1994. Water-soluble protein, cartilage, extracellular matrix are hot keywords, indicating that water-soluble protein and extracellular matrix as a carrier of bone materials to induce cartilage formation and differentiation was a hot research direction.

According to the latest keywords in each cluster, after 2018, proliferation, osteogenic differentiation, stromal cells, delivery, and mechanical properties have became new terms, which means that in the future, drug delivery, vascularization, and osteogenic differentiation of bone defects, and the performance of biomaterials will become a new research direction and continue to be discussed in depth.

## Conclusion

This study uses CiteSpace to reveal the dynamic changes, leaders, and cooperating institutions in the field of bone defects since 1994 and provides references for new technologies and in-depth research directions. The annual publications of bone defects increased exponentially. The high-yield author was Professor Choi Seong-Ho at Yonsei University in South Korea. The Sao Paulo University and the Chinese Academy of Sciences were potential cooperative institutions. The drug delivery, vascularization, osteogenic differentiation and biomaterial properties of bone defects are expected to be further studied.

## Supplementary information

**Additional file 1.** Supplementary Table 1 Co-cited frequency and centrality of countries (regions) and institutions..

**Additional file 2.** Supplementary Table 2 Top 5 jornals in terms of co-cited frequency or centrality

**Additional file 3.** Supplementary Table 3 Top 10 keywords in terms of co-cited frequency and centrality.

## Data Availability

The datasets used and analyzed during the current study are available from the corresponding author on reasonable request.

## Abbreviations

IF: Impact factor
RHBMP 2: Recombinant human bone morphogenetic protein 2
UIWON JUNG: Jung Ui-Won
CK KIM: Kim Chong-Kwan
AHMAD ORYAN: Oryan Ahmad
SH CHOI: Choi Seong-Ho
JOHN A JANSEN: Jansen John A
KS CHO: CHO KS
CS KIM: Kim Chang-Sung
YK LEE: Lee Yong-Keun
UME WIKESJO: Wikesjoe Ulf M. E.
Univ: University
PEOPLES R CHINA: People’s Republic of China
Chinese Acad of Sci: Chinese Academy of Sciences
Clin Orthop Relat R: Clinical Orthopaedics and Related Research
J Bone Joint Surg AM: Journal of Bone and Joint Surgery-American Volume
J Biomed Mater Res A: Journal of Biomedical Materials Research Part A
Clin Orthop Relat R: Clinical Orthopaedics and Related Research
J Periodontol: Journal of Periodontology
LLR: Likelihood Ratio
