# Supplementary material for: Research hotspots and trends of bone defects based on Web of Science: a bibliometric analysis
Source: J Orthop Surg Res. 2020 Oct 8;15:463. doi: 10.1186/s13018-020-01973-3 (PMC7545570; doi:10.1186/s13018-020-01973-3)
Supplement: Supplementary file 1 — Additional file 1. Supplementary Table 1 Co-cited frequency and centrality of countries (regions) and institutions.. [file 13018_2020_1973_MOESM1_ESM.docx]

**Supplementary Table 1. Co-cited frequency and centrality of countries (regions) and institutions.**

| **Co-cited frequency** | **Centrality** | **Countries (Regions) and Institutions** |
| --- | --- | --- |
| 1169 | 1.1 | USA |
| 824 | 0.1 | People's Republic of China |
| 480 | 0.26 | Germany |
| 452 | 0.2 | Japan |
| 319 | 0.04 | South Korea |
| 198 | 0.17 | Italy |
| 39 | 0.17 | Finland |
| 93 | 0 | Shanghai Jiaotong University |
| 83 | 0.03 | Sao Paulo University |
| 60 | 0 | Sichuan University |
| 41 | 0 | Yonsei University |
| 39 | 0.01 | Harvard University |
| 20 | 0.01 | Radboud University Nijmegen |
| 13 | 0.01 | Bernese University |
| 8 | 0.01 | University of Dusseldorf |
