# Supplementary material for: Research hotspots and trends of bone defects based on Web of Science: a bibliometric analysis
Source: J Orthop Surg Res. 2020 Oct 8;15:463. doi: 10.1186/s13018-020-01973-3 (PMC7545570; doi:10.1186/s13018-020-01973-3)
Supplement: Supplementary file 2 — Additional file 2. Supplementary Table 2 Top 5 jornals in terms of co-cited frequency or centrality [file 13018_2020_1973_MOESM2_ESM.docx]

**Supplementary Table 2. Top 5 jornals in terms of co-cited frequency or centrality.**

| **Co-cited frequency** | **Centrality** | **Journals** |
| --- | --- | --- |
| 2091 | 0.84 | Clinical Orthopaedics and Related Research |
| 1845 | 0.56 | Biomaterials |
| 1806 | 0.29 | Journal of Bone and Joint Surgery-American Volume |
| 1095 | 0.22 | Bone |
| 781 | 0 | Journal of Biomedical Materials Research Part A |
| 342 | 0.28 | Journal of Periodontology |
