# Supplementary material for: Research hotspots and trends of bone defects based on Web of Science: a bibliometric analysis
Source: J Orthop Surg Res. 2020 Oct 8;15:463. doi: 10.1186/s13018-020-01973-3 (PMC7545570; doi:10.1186/s13018-020-01973-3)
Supplement: Supplementary file 3 — Additional file 3. Supplementary Table 3 Top 10 keywords in terms of co-cited frequency and centrality. [file 13018_2020_1973_MOESM3_ESM.doc]

**Supplementary Table 3. Top 10 keywords in terms of co-cited frequency and centrality.**

| **Co-cited frequency** | **Centrality** | **Keywords** |
| --- | --- | --- |
| 757 | 0.44 | regeneration |
| 508 | 0.29 | repair |
| 381 | 0.39 | in vitro |
| 360 | 0.25 | bone regeneration |
| 347 | 0.01 | mesenchymal stem cell |
| 306 | 0 | scaffold |
| 233 | 0.2 | reconstruction |
| 156 | 0.16 | graft |
| 107 | 0.05 | bone defect |
| 105 | 0.03 | implant |
| 52 | 0.14 | guided tissue regeneration |
| 49 | 0.26 | hydroxyapatite |
| 25 | 0.35 | cell |
| 14 | 0.14 | rabbit |
